# Protective Effect of* Psoralea corylifolia* L. Seed Extract against Palmitate-Induced Neuronal Apoptosis in PC12 Cells

**DOI:** 10.1155/2016/5410419

**Published:** 2016-10-23

**Authors:** Yunkyoung Lee, Hee-Sook Jun, Yoon Sin Oh

**Affiliations:** ^1^College of Pharmacy and Gachon Institute of Pharmaceutical Science, Gachon University, Incheon 406-840, Republic of Korea; ^2^Lee Gil Ya Cancer and Diabetes Institute, Gachon University, Incheon 406-840, Republic of Korea; ^3^Gachon Medical Research Institute, Gil Hospital, Incheon 405-760, Republic of Korea

## Abstract

The extract of* Psoralea corylifolia* seeds (PCE) has been widely used as a herbal medicine because of its beneficial effect on human health. In this study, we investigated the protective effects and molecular mechanisms of PCE on palmitate- (PA-) induced toxicity in PC12 cells, a neuron-like cell line. PCE significantly increased cell viability in PA-treated PC12 cells and showed antiapoptotic effects, as evidenced by decreased expression of cleaved caspase-3, cleaved poly(ADP-ribose) polymerase, and bax protein as well as increased expression of bcl-2 protein. In addition, PCE treatment reduced PA-induced reactive oxygen species production and upregulated mRNA levels of antioxidant genes such as nuclear factor (erythroid-derived 2)-like 2 and heme oxygenase 1. Moreover, PCE treatment recovered the expression of autophagy marker genes such as beclin-1 and p62, which was decreased by PA treatment. Treatment with isopsoralen, one of the major components of PCE extract, also recovered the expression of autophagy marker genes and reduced PA-induced apoptosis. In conclusion, PCE exerts protective effects against lipotoxicity via its antioxidant function, and this effect is mediated by activation of autophagy. PCE might be a potential pharmacological agent to protect against neuronal cell injury caused by oxidative stress or lipotoxicity.

## 1. Introduction

Neuronal apoptosis occurs in diabetic patients [1] and diabetic animal models [2], suggesting that neuronal injury plays a role in the development of diabetic complications such as neuropathy [3]. Chronic hyperglycemia (glucotoxicity) and hyperlipidemia (lipotoxicity) cause apoptosis in various kinds of neuronal cells (Schwann cells, PC12 cells, and cortical cells) [4, 5], which results in neuronal dysfunction such as impairment of learning and memory abilities and cognitive deficits.

Reactive oxygen species (ROS) production by oxidative stress is the main cause of neuronal cell death by high glucose or lipid toxicity [6, 7]. High glucose increases oxidative stress via pathways involving reactive oxygen intermediates, and free fatty acids activate nicotinamide adenine dinucleotide phosphate oxidase, which leads to oxidative stress [7, 8]. Compared to other parts of our body, the central nervous system is more sensitive to oxidative stress due to its high oxygen consumption and lipid content [9]. Therefore, antioxidants may have a positive effect in the central nervous system and may be a promising approach for neuroprotection therapy.

Recent studies have shown that oxidative stress and ROS regulate autophagy. Cellular accumulation of ROS stimulates autophagy in various cells including neuronal cells [10–12], and increased autophagy reduces oxidative damage via degradation of oxidized biomolecules (proteins, DNA, and lipids) through an autophagosomal-lysosomal pathway [13]. However, chronic exposure to oxidative stress reduces autophagic activity [14] and consequently programed cell death occurs. Autophagy is also known to play an important role in a variety of neurodegenerative conditions, including Alzheimer's disease, Parkinson's disease, and Huntington's disease [15–17] but has not been well studied in diabetic neuropathy [18].

Extract of* Psoralea corylifolia* seeds (PCE), commonly known as “Boh-Gol-Zhee” in Korea, is a widely used medicinal preparation and shows antibacterial, antitumor, and antioxidant effects [19–21]. PCE contains a number of chemical compounds, such as coumarins (including psoralidin, psoralen, and isopsoralen) and meroterpenes (including bakuchiol and 3,2-hydroxybakuchiol). PCE or its single compounds show neuroprotective effects against cytotoxic insults such those caused by as 1-methyl-4-phenylpyridinium or 3-nitropropionic acid [22, 23], but its possible neuroprotective effects against glucotoxicity or lipotoxicity have not been studied. Therefore, we investigated the protective effect of PCE against palmitate- (PA-) induced lipotoxicity in rat pheochromocytoma PC12 cells and investigated the mechanisms involved in the antilipotoxic effect of PCE.

## 2. Materials and Methods

### 2.1. Materials

RPMI-1640 medium (11 mM glucose and L-glutamine) and fetal bovine serum were purchased from Gibco (Paisley, UK). Penicillin/streptomycin antibiotic mixture and Dulbecco's phosphate-buffered saline (DPBS) were purchased from WELGENE (Daegu, Korea). 3-(4,5-Dimethylthiazolyl-2)-2,5-diphenyltetrazolium bromide (MTT) was obtained from Duchefa (Haarlem, Netherlands). Primary antibodies against poly(ADP-ribose) polymerase (PARP) (9542), caspase-3 (9662), bcl-2 (2876), bax (2772), p62 (5224S), and beclin-1 (3495) were purchased from Cell Signaling Technology (Beverly, MA, USA), and horseradish peroxidase-conjugated secondary antibodies (anti-rabbit, sc-2004; anti-mouse, sc-2005) were obtained from Santa Cruz Biotechnology (Santa Cruz, CA, USA). Psoralen, isopsoralen, palmitate, rapamycin, and bovine serum albumin were obtained from Sigma-Aldrich (St Louis, MO). Bakuchiol was purchased from Enzo Life Sciences Inc. (Farmingdale, NY).

### 2.2. Preparation of PCE

*Psoralea corylifolia* seeds were purchased from an oriental drug store (Kwang Myung Dang Co., Ulsan, Korea), and the water extract was prepared as described previously [24, 25]. In brief, the dried seeds (300 g) were ground into small pieces and then extracted with distilled water under reflux two times. The combined water extract was evaporated* in vacuo* to obtain a dark brownish residue (61.92 g).

### 2.3. PCE Treatment

PCE was used at concentrations of 1, 10, 30, 50, or 100 *μ*g/ml, and we found that the concentration of 50 *μ*g/ml was most effective. PC12 cells were pretreated with 50 *μ*g/ml PCE in culture medium for 2~6 h and then 0.4 mM palmitate was added with 50 *μ*g/ml PCE for various times as indicated in the figures.

### 2.4. Cell Culture

PC12 rat pheochromocytoma cells were obtained from the Korea Cell Line Bank at Seoul National University (Seoul, Korea). Cells were maintained in RPMI-1640 medium containing 10% fetal bovine serum and 1% penicillin/streptomycin at 37°C in humidified atmosphere of 5% CO_2_. Cells were subcultured at a ratio of 1 : 3–1 : 4 when the cultures were 75–80% confluent, and the medium was changed every 2 days.

### 2.5. Preparation of Palmitate

PA (Sigma, P9767) was conjugated to fatty acid free bovine serum albumin (FAF-BSA; Sigma, A0281). PA (51.2 mg) was dissolved in 10 ml DPBS at 60°C for 20 min to make a 20 mM stock solution, and the pH was adjusted with 1 M NaOH. FAF-BSA was dissolved in DPBS and sterilized by filtering through a sterile membrane filter. PA (20 mM) was complexed in a 1 to 3 molar ratio with 5% FAF-BSA to generate a 5 mM PA stock solution, and then PA was diluted in RPMI-1640 medium to make a 0.4 mM PA working solution. 0.3% FAF-BSA media was used as a control.

### 2.6. MTT Assay

Cell viability was evaluated by MTT assay. Briefly, the cells were plated in 96-well culture plates at a density of 2.5 × 10^4^ cells/well. After 24 h, the cells were incubated with 400 or anticytotoxic substances for 24–48 h. Thereafter, after 24–48 h of treatment, 100 *μ*l/well MTT (500 *μ*g/ml MTT in medium) was added, and the cells were incubated for an additional 1 h at 37°C. After removing the MTT, the cells and dye crystals were solubilized by adding 100 *μ*l isopropanol. The absorbance of each sample at a wavelength of 570 nm was detected using a microplate spectrophotometer. The cell viability in response to treatment was calculated as percentage of control cell viability = (OD treated-cells/OD control cells) × 100.

### 2.7. Annexin V and Propidium Iodide Staining

Apoptosis was detected using an Annexin V fluorescein isothiocyanate (FITC)/propidium iodide (PI) apoptosis detection kit (BD Biosciences, Franklin Lakes, NJ, USA) according to the manufacturer's instruction. Briefly, the cells were plated in 12-well culture plates at the density of 1 × 10^5^ cells/well. After treatment, cells were trypsinized and collected together with floating dead cells. Cells were washed twice with cold phosphate buffered saline and then resuspended in 1x binding buffer. Annexin V-FITC and PI were added into the tube and incubated for 15 min at room temperature in the dark. Then, cells were analyzed using flow cytometry (FACS Calibur, Becton Dickinson, CA, USA). The Annexin V(−)/PI(−) population was regarded as normal healthy cells. Apoptotic cells were expressed as a percentage of the total number of cells.

### 2.8. Quantitative Real-Time Polymerase Chain Reaction (RT-PCR)

The total RNA was extracted from the cultured cells using TRIZOL reagent (Invitrogen Corp., Carlsbad, CA, USA) following the manufacturer's instructions, and cDNA was synthesized using a PrimeScript 1st strand cDNA synthesis kit (Takara Bio Inc., Kyoto, Japan). Quantitative RT-PCR was performed using the SYBR Premix Ex Taq II, ROX plus (Takara), and the Prism 7900HT sequence detection system (Applied Biosystems, Foster City, CA, USA). PCR was carried out for 40 cycles (2 min at 50°C, 10 min at 95°C, and 40 cycles of 10 s at 95°C and 1 min at 60°C). The relative copy number was calculated using the threshold crossing point (Ct) as calculated by ΔΔCt. The primer sequences used in this study are indicated in Table 1.

### 2.9. Western Blotting

PC12 cells were harvested and lysed using lysis buffer with a mixture of protease inhibitors. Total proteins were extracted and quantified using the Bradford assay. Subsequently, 25 *μ*g of protein was fractionated using sodium dodecyl sulfate polyacrylamide gel electrophoresis and electrotransferred onto nitrocellulose membranes. Membranes were blocked with 3% skimmed milk in Tris-buffered saline containing 0.1% Tween-20 and then incubated overnight with the following primary antibodies diluted in 3% skimmed milk at 4°C: anti-PARP, anti-caspase-3, anti-bcl-2, anti-bax, anti-p62, anti-beclin-1 (all 1 : 1,000 dilution), or anti-actin (1 : 5,000 dilution). The blots were washed and incubated with a horseradish peroxidase-conjugated secondary antibody (all 1 : 2,000 dilution) for 2 h at room temperature. The blots were developed using the ECL detection system (Millipore, Billerica, MA, USA), and a LAS-4000 mini system (Fujifilm Corp., Tokyo, Japan) was used for visualization. The intensities of the bands were normalized to the actin band using Image J software (National Institutes of Health, Bethesda, MD).

### 2.10. Measurement of ROS

The formation of ROS was evaluated by the fluorogenic dye 2′,7′-dichlorodihydrofluorescein diacetate (H_2_-DCFDA, Invitrogen, Carlsbad, CA, USA), which is oxidized by intracellular ROS. Cells (1 × 10^4^ cells/well) were seeded in black 96-well plates and pretreated with substances, and then cells were incubated with 10 *μ*M H_2_-DCFDA for 30 min. The cells were washed with phosphate buffered saline, and the resulting fluorescent compound (2′,7′-dichlorofluorescein, DCF) was detected by a fluorescence microplate reader with an excitation wavelength of 485 nm and an emission wavelength of 530 nm. The data for the treatment groups were expressed as a percentage of the fluorescence generated by the control.

### 2.11. Statistical Analysis

Values are expressed as the mean ± standard error of the mean (SEM) of three independent experiments. Data were analyzed using Analysis of Variance followed by* post hoc* analyses using the Tukey range test (SPSS 10.0 statistical software, SPSS Inc., Chicago, IL, USA). *P* < 0.05 was considered to indicate a statistically significant difference between values.

## 3. Results

### 3.1. Protective Effect of PCE on PA-Induced Toxicity in PC12 Cells

**Figure 1:**
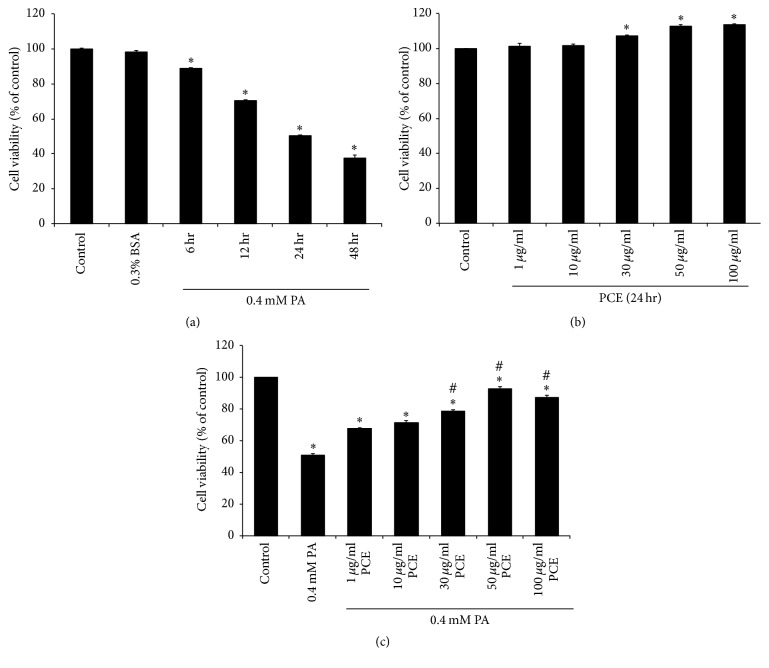
Effect of PCE on PA-induced toxicity in PC12 cells. (a) PC12 cells were treated with PA (0.4 mM) for the indicated times. (b) Cells were incubated in media containing various concentrations of PCE (1, 10, 30, 50, and 100 *μ*g/ml) for 24 h. (c) Cells were preincubated with 1–100 *μ*g/ml PCE for 6 h, followed by exposure to 0.4 mM PA for 24 h. Cell viability was determined by MTT assay. The results represent the mean ± SEM from triplicate experiments and were normalized to control cells. ^*∗*^ *P* < 0.001 as compared with control. ^#^ *P* < 0.001 as compared with PA.

To check the impact of lipotoxicity on PC12 cell viability, cells were incubated with 0.4 mM PA for 6 to 48 h. The MTT assay showed that cell viability was significantly reduced after 6 h of treatment with PA, and 50.4% cell viability was observed after 24 h of PA treatment compared with control cells (Figure 1(a)). When we treated PC12 cells with 1–100 *μ*g/ml PCE for 24 h, no cytotoxic effects were observed, and 30–100 *μ*g/ml PCE treatment significantly increased cell viability (Figure 1(b)). To examine the effect of PCE on PA-treated cell cytotoxicity, cells were pretreated with PCE (1–100 *μ*g/ml) for 6 h and then 0.4 mM was added. 1 and 10 *μ*g/ml PCE had no effect on lipotoxicity, but pretreatment with 30–100 *μ*g/ml PCE significantly increased cell viability in the presence of PA compared with cells with no PCE pretreatment. The protective effect of PCE against lipotoxicity was most effective at 50 *μ*g/ml PCE (Figure 1(c)).

### 3.2. Antiapoptotic Effect of PCE against Lipotoxicity

**Figure 2:**
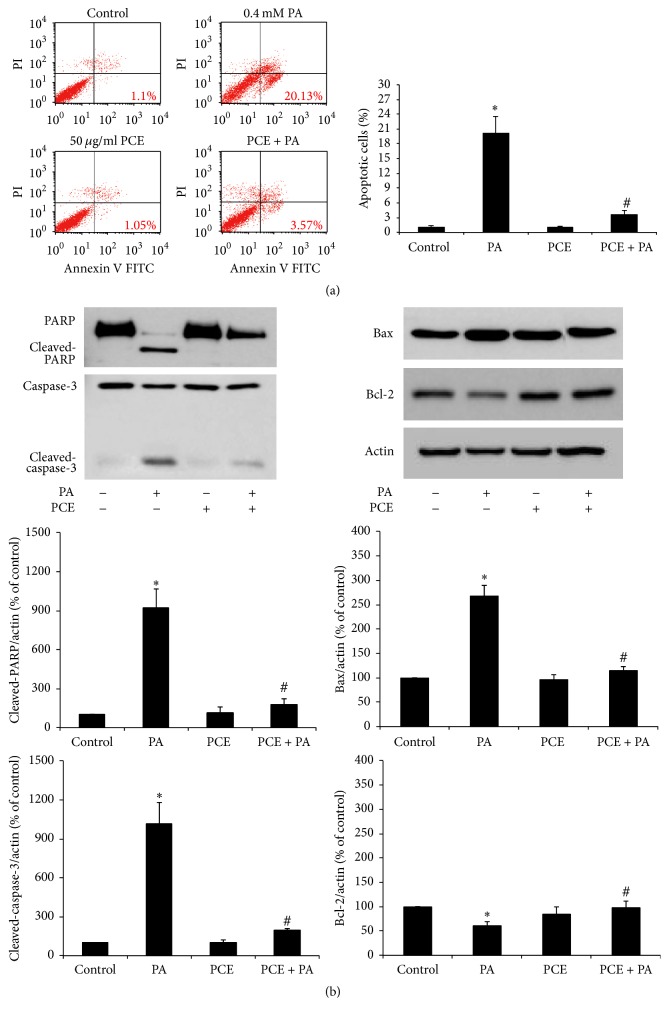
Effect of PCE on PA-induced apoptosis in PC12 cells. PC12 cells were pretreated with 50 *μ*g/ml PCE for 6 h, followed by exposure to 0.4 mM PA for 24 h. (a) The cells were costained with FITC-Annexin V/PI and analyzed by flow cytometry to determine the population of cells in early apoptosis. Graph in the right side shows quantitative data for the percentage of early apoptotic cells (the lower right quadrant) according to treatment. (b) Expression levels of apoptosis-related proteins. Western blot analyses were performed to examine the expression of PARP, caspase-3, bax, and bcl-2. Actin was used as the internal control. The bands were quantified by Image J software. The results represent the mean ± SEM from triplicate experiments. ^*∗*^ *P* < 0.01 as compared with control. ^#^ *P* < 0.05 as compared with PA.

To determine whether PCE protects PC12 cells from PA-induced apoptosis, we performed FITC-Annexin V and PI staining after PA treatment with or without PCE pretreatment. As shown in Figure 2(a), the proportion of Annexin V-FITC^+^/PI^−^-cells was increased by PA treatment, and 50 *μ*g/ml PCE pretreatment significantly inhibited this increase (control, 1.1 ± 0.3%; 0.4 mM PA, 20.1 ± 3.5%; 50 *μ*g/ml PCE, 1.1 ± 0.4%; PCE + PA, 3.6 ± 0.9%). Western blot analysis of apoptosis-related proteins showed that PA treatment increased the expression levels of proapoptotic proteins such as bax, cleaved-caspase-3, and cleaved-PARP, and PCE pretreatment markedly reduced the expression of these proteins. PCE alone did not significantly change the expression levels of these proteins compared with control. The expression level of bcl-2, a prosurvival marker, was decreased by PA, and it was increased by PCE pretreatment (Figure 2(b)).

### 3.3. Antioxidant Effect of PCE on Lipotoxicity-Induced ROS Generation

**Figure 3:**
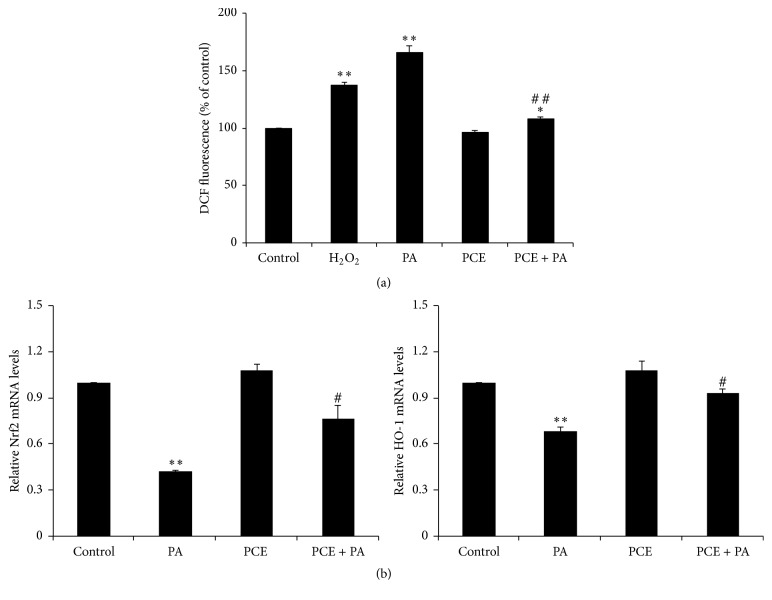
Effect of the PCE on PA-induced oxidative stress in PC12 cells. (a) PC12 cells were pretreated with 50 *μ*g/ml PCE for 2 h, followed by exposure to 0.4 mM PA for 3 h. The cells were stained with 10 *μ*M H_2_-DCFDA, and intracellular ROS generation was determined by DCF. H_2_O_2_ (200 *μ*M) was used as a positive control. (b) The cells were pretreated with 50 *μ*g/ml PCE for 6 h, followed by exposure to 0.4 mM PA for 24 h. The mRNA levels of Nrf2 and HO-1 were analyzed by quantitative RT-PCR. The mRNA levels were normalized with those of cyclophilin. The results shown represent the mean ± SEM from triplicate experiments. ^*∗*^ *P* < 0.05 and ^*∗∗*^ *P* < 0.001 as compared with control. ^#^ *P* < 0.05 and ^##^ *P* < 0.001 as compared with PA.

To investigate the effects of PCE on ROS production in PA-treated PC12 cells, we measured the intracellular ROS level as assessed by DCF fluorescence intensity after treatment with 0.4 mM PA in the presence or absence of PCE pretreatment. Exposure to PA alone induced a significant increase in DCF fluorescence levels, similar to the results obtained with H_2_O_2_-treated cells, and this increase was prevented by pretreatment with 50 *μ*g/ml PCE (Figure 3(a)). To determine whether the inhibitory effect of PCE on PA-induced oxidative stress was associated with the induction of antioxidant genes, we measured the mRNA expression levels of nuclear factor-like 2 (Nrf2) and heme oxygenase (HO) 1 using quantitative RT-PCR. A decrease of Nrf2 and HO-1 mRNA expression by PA treatment was observed, and the decrease was significantly reversed by PCE treatment (Figure 3(b)).

### 3.4. Involvement of Autophagy in Protective Effect of PCE against Lipotoxicity

**Figure 4:**
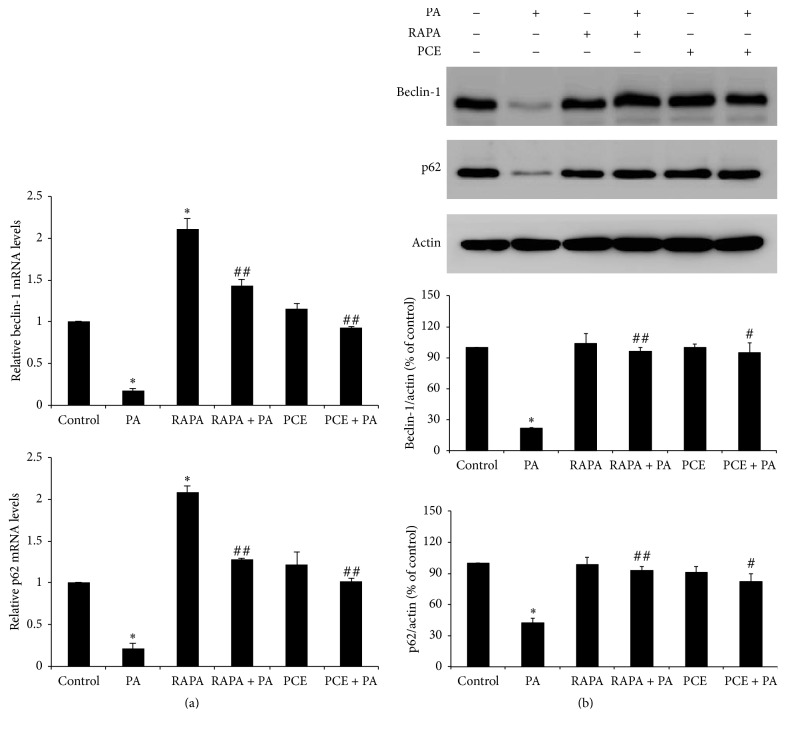
Effect of PCE on PA-induced autophagy in PC12 cells. PC12 cells were pretreated with 1 *μ*M of rapamycin (RAPA) for 2 h (as a positive control) or 50 *μ*g/ml PCE for 6 h, followed by exposure to 0.4 mM PA for 24 h. (a) Total RNA was extracted from PC12 cells, and the mRNA expression levels of beclin-1 and p62 were analyzed by quantitative RT-PCR. The mRNA levels were normalized with those of cyclophilin. (b) Protein levels of beclin-1 and p62 were analyzed by western blot. Actin was used as the internal control. The bands were quantified by Image J software. The results shown represent the mean ± SEM from triplicate experiments. ^*∗*^ *P* < 0.001 as compared with control. ^#^ *P* < 0.01 and ^##^ *P* < 0.001 as compared with PA.

As autophagy is involved in neuronal cell survival [26], we measured the expression level of autophagy marker genes, beclin-1 and p62, which play a role in autophagosome formation and lysosomal fusion [27]. The mRNA (Figure 4(a)) and protein (Figure 4(b)) levels of autophagy marker genes were reduced in PA-treated cells compared with control cells, and this was reversed by PCE treatment. To confirm whether induction of autophagy participates in PCE's cytoprotective effect, cells were treated with rapamycin, an autophagy enhancer. As shown in Figure 4, rapamycin treatment induced p62 and beclin-1 mRNA expression and significantly increased both mRNA and protein levels of beclin-1 and p62 in PA-treated cells, which was similar to that of PCE-pretreated cells.

As it was reported that autophagy is regulated by oxidative stress [28], we further evaluated the correlation between autophagy and oxidative stress on lipotoxic neuronal apoptosis. Cells were treated with 0.5 mM* N*-acetyl-*L*-cysteine (NAC), a ROS scavenger, and the expression level of autophagy marker genes was measured. As shown in Figure S1 in Supplementary Material available online at http://dx.doi.org/10.1155/2016/5410419, PA treatment induced high DCF fluorescence, which was reduced by NAC (Figure S1A). Similarly, the expression level of beclin-1 and p62 was decreased by PA treatment, which was reversed by NAC (Figure S1B).

### 3.5. Antiapoptotic Effect of Isopsoralen on PA-Induced Apoptosis

**Figure 5:**
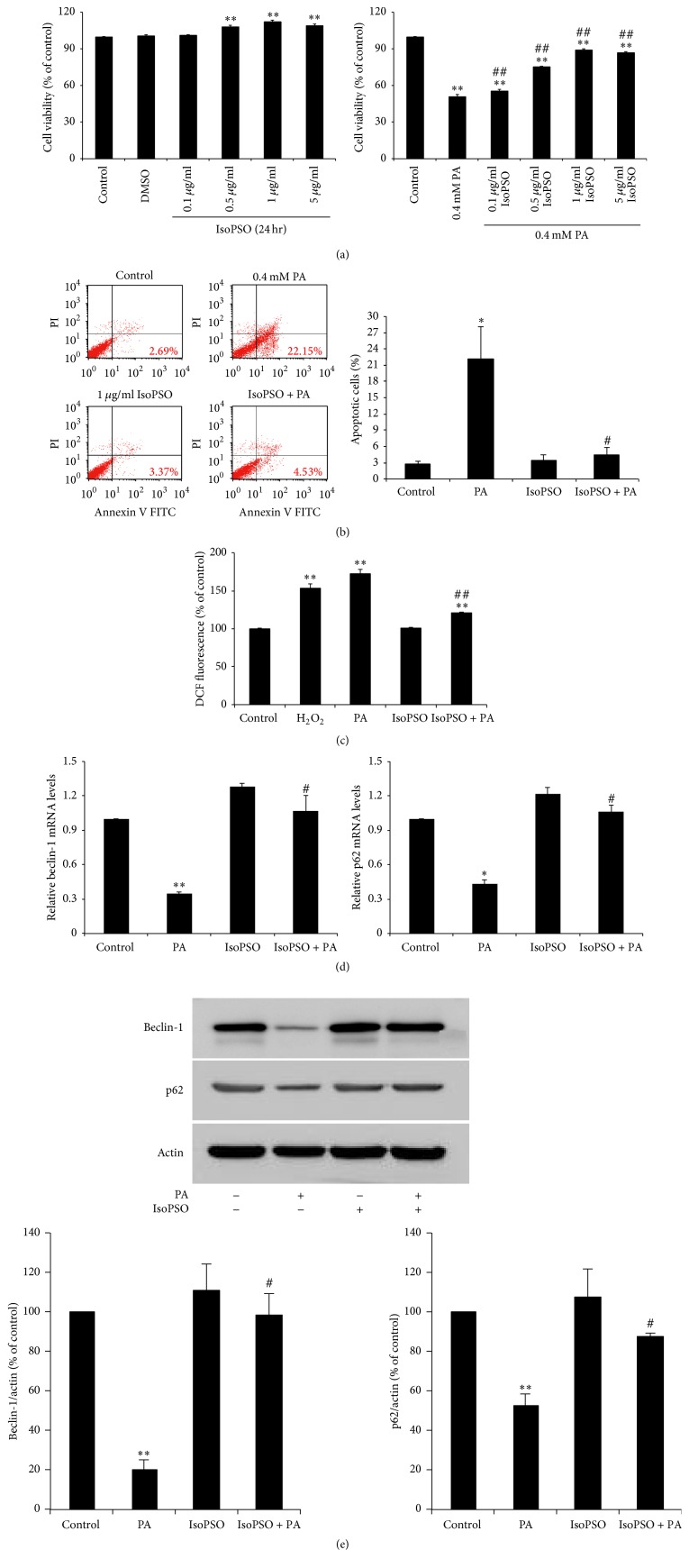
Effect of IsoPSO on PA-induced apoptosis, ROS production, and autophagy in PC12 cells. (a) PC12 cells were incubated in media containing isopsoralen (IsoPSO) (0.1–5 *μ*g/ml) for 24 h (left side). The cells were pretreated with 0.1–5 *μ*g/ml IsoPSO for 6 h, followed by exposure to 0.4 mM PA for 24 h (right side). Cell viability was measured by MTT assay. (b) The cells were pretreated with 1 *μ*g/ml IsoPSO for 6 h, followed by exposure to 0.4 mM PA for 24 h. The cells were costained with FITC-Annexin V/PI and analyzed by flow cytometry to determine the population of cells in the early stage of apoptosis. Graph shows quantitative data for the percentage of early apoptotic cells (the lower right quadrant). (c) Cells were pretreated with 1 *μ*g/ml IsoPSO for 2 h, followed by exposure to 0.4 mM PA for 3 h. The cells were stained with 10 *μ*M H_2_-DCFDA, and intracellular ROS generation was determined by DCF. H_2_O_2_ (200 *μ*M) was used as a positive control. (d) The cells were pretreated with 1 *μ*g/ml IsoPSO for 6 h, followed by exposure to 0.4 mM PA for 24 h. Total RNA was extracted from PC12 cells and the mRNA expression levels of beclin-1 and p62 were analyzed by quantitative RT-PCR. The mRNA levels were normalized with those of cyclophilin. (e) Protein levels of beclin-1 and p62 were analyzed by western blot analyses. Actin was used as the internal control. The results shown represent the mean ± SEM from triplicate experiments. ^*∗*^ *P* < 0.05 and ^*∗∗*^ *P* < 0.001 as compared with control. ^#^ *P* < 0.05 and ^##^ *P* < 0.001 as compared with PA.

As bakuchiol, psoralen, and isopsoralen are known to be major constituents of PCE, we investigated whether any of these compounds are responsible for the protective effect of PCE in PA-treated PC12 cells. Treatment with bakuchiol at concentrations from 0.01 to 5 *μ*g/ml increased cell viability. Psoralen (10 *μ*g/ml) and isopsoralen (1–10 *μ*g/ml) also significantly increased cell viability, but 50 *μ*g/ml of either of these components reduced cell viability compared with control cells (Figure S2A). The MTT assay showed that treatment with psoralen or isopsoralen, but not bakuchiol, markedly increased cell viability of PA-treated cells (Figure S2B). As we found that isopsoralen showed the higher antioxidant activity compared with psoralen (unpublished data), we examined the antiapoptotic effects of isopsoralen on PA-induced apoptosis. Cell viability was significantly increased by 0.5–5 *μ*g/ml isopsoralen (Figure 5(a), left side) and pretreatment with isopsoralen reduced PA-induced cytotoxicity (Figure 5(a), right side). Apoptotic cells (Figure 5(b)) and ROS overproduction (Figure 5(c)) by PA treatment was also inhibited after pretreatment with 1 *μ*g/ml isopsoralen. Finally, we confirmed that isopsoralen induced p62 and beclin-1 mRNA (Figure 5(d)) and protein (Figure 5(e)) expression in PA-treated PC12 cells.

## 4. Discussion

Hyperlipidemia occurs during the progression of type 2 diabetes, and lipid profiles are abnormal in patients with diabetic neuropathy, suggesting that hyperlipidemia may be one of the major factors in the development of diabetic neuropathy. Increased levels of free fatty acids can induce neuronal cell apoptosis [4] and oxidize low density lipoproteins, which also cause injury to neuronal cells via the oxidative stress pathway [29]. Clinical evidence shows that hyperlipidemia-induced oxidative stress predisposes diabetic patients to complications and its inhibition may block the initiation and progression of diabetic neuropathy [30].

PCE is a well-known traditional Chinese herbal medicine and has antioxidant activity. Previously, we found that PCE has protective effects against high fat diet-induced inflammation in the liver and H_2_O_2_-induced apoptosis in beta cells [24, 31], suggesting that PCE might have ameliorative effects on diabetic complications. Neuroprotective effects of PCE against damage caused by neurotoxic agents, such as 3-nitropropionic acid and 1-methyl-4-phenylpyridinium [22, 23], have been previously reported, but the effect on neuronal cell death caused by glucotoxicity or lipotoxicity has not been studied. In this study, we investigated the protective effect of PCE against lipotoxicity, which is physiologically relevant, using neuron-like PC12 cells.

In this study, PA induced apoptotic death in PC12 cells as evidenced by induction of apoptosis markers such as the increase of Annexin V^+^ cells and the increased expression of bax, cleaved-caspase-3, and cleaved-PARP proteins, which is consistent with a previous study [32]. Interestingly, PCE pretreatment reduced the expression of PA-induced apoptotic proteins and the Annexin V^+^ cell population, suggesting that PCE has antiapoptotic effects under conditions of lipotoxicity.

Although the exact mechanism of lipotoxicity is currently unknown, the increased production of ROS has been suggested as one of the major causes of PA-induced apoptosis [32]. Some studies demonstrated that ROS scavenger treatment decreased amyloid *β*-induced or high glucose-induced neuronal cell apoptosis in U87, SH-SY5Y, and PC12 cells [33, 34]. We found that PCE treatment attenuated ROS overproduction and decreased Nrf2 and HO-1 mRNA expression induced by PA, suggesting that PCE has antioxidant effects in lipotoxic conditions. Similarly, Kim et al. reported that compounds isolated from PCE showed antioxidant effects against oxidative stress-induced retinal damage [35].

Previous studies reported that fatty acid-induced lipotoxicity was associated with autophagy in hepatocytes, beta cells, and endothelial cells [36–38]. Autophagy, a ubiquitous catabolic pathway involved in both cell survival and cell death, has been implicated in the pathogenesis of major neurodegenerative disorders [39]. Recent findings have shown that autophagy plays a crucial role for neuronal function and cell survival. Heterozygous deletion of beclin-1 in mice decreases neuronal autophagy and results in neurodegeneration [15], and transgenic mice lacking autophagy-related 5 (Atg5) specifically in neural cells develop progressive deficits in motor function and abnormal protein accumulation [40]. Meng et al. reported that rapamycin, an autophagy enhancer, treatment ameliorates thiamine deficiency (neuronal oxidative stressor) induced apoptotic cell death both in SH-SY5Y cells and the submedial thalamus in mice [41]. In addition, upregulation of autophagy marker gene expression by treatment with quercetion also alleviates high glucose induced Schwann cell injury [42]. In this study, we observed a decreased expression of autophagy marker genes (beclin-1 and p62) in response to PA treatment, and treatment with PCE reversed this decrease. In agreement with this, treatment with rapamycin recovers the expression of p62 and beclin-1, and the level was similar to the levels observed in cells treated with PA after addition of PCE. These results suggest that increased autophagy is involved in PCE's protective effect against lipotoxicity.

It has been reported that autophagy was correlated with oxidative stress signaling [28]. We found that, after inhibition of ROS generation using NAC treatment, PA fail to reduce p62 and beclin-1 in PC12 cells and these results demonstrated that NAC ameliorated autophagy inhibition which occurred during lipotoxicity induced neuronal injury. Jain et al. reported that p62 is a target for Nrf2 [43] and enhanced expression level of autophagy marker genes by PCE treatment suggesting that autophagy activation was involved in antioxidant effect of PCE to prevent lipotoxicity.

Bakuchiol, psoralen, and isopsoralen are major compounds isolated from PCE, and we found that pretreatment with psoralen or isopsoralen, which are both coumarins, inhibited neuronal cell death in PA-treated PC12 cells. We found that treatment with isopsoralen also restored beclin-1 and p62 levels in PA-treated cells similar to PCE treatment, suggesting that the antilipotoxic effect of PCE was mediated by isopsoralen. Similarly, our previous study found that psoralen and isopsoralen inhibited apoptotic cell death in H_2_O_2_-treated INS-1 cells [24]. Although bakuchiol exerts protective effects against H_2_O_2_-induced oxidative stress in hepatocytes [25], we did not observe a protective effect against PA-induced oxidative stress in PC12 cells. These results suggest that the antiapoptotic effect of bakuchiol may be cell-specific, and different mechanisms may be involved to reduce oxidative stress by H_2_O_2_ as compared with PA.

## 5. Conclusion

In conclusion, we showed an ameliorative effect of PCE on PA-induced cell injury in PC12 cells. PCE treatment decreased the lipotoxic effect via antioxidant function, and autophagy activation was involved in the antiapoptotic action of PCE. In addition, isopsoralen and psoralen are active components of PCE with respect to neuronal cell protection. These results provide insights into the therapeutic potential of PCE and isopsoralen against lipotoxicity-induced neuronal cell death.

## Supplementary Material

Supplementary Figure 1 showed a correlation between reactive oxygen species production and autophagic activity in PA-treated PC12 cells.
Supplementary Figure 2 showed the effects of PCE single compounds on PA-induced toxicity of PC12 cells.

## Figures and Tables

**Table 1:** Primers used for quantitative real-time PCR. F, forward; R, reverse.

Primers	Sequences
Cyclo	F: 5′-GGT CTT TGG GAA GGT GAA AGA A-3′
	R: 5′-GCC ATT CCT GGA CCC AAA A-3′
Bax	F: 5′-AGA CAC CTG AGC TGA CCT TGG A-3′
	R: 5′-CGG AGA CAC TCG CTC AGC TT-3′
Bcl-2	F: 5′-GGG ATG CCT TTG TGG AAC TAT ATG-3′
	R: 5′-CAG CCA GGA GAA ATC AAA CAG A-3′
Nrf-2	F: 5′-GAG ACG GCC ATG ACT GAT-3′
	R: 5′-GTG AGG GGA TCG ATG AGT AA-3′
HO-1	F: 5′-GTG AGA AGA GCC CTG ATT GT-3′
	R: 5′-CCT GTG ATG TCG TTT CTG GA-3′
Beclin-1	F: 5′-AGT GGA CAA AGG CGC TCA A-3′
	R: 5′-CCC AAG CAA GAC CCC ACT T-3′
p62	F: 5′-ATG GAG CCG GAG AAT AAG TAC CT-3′
	R: 5′-CGG GTC GAG CGA GTC CTT-3′
