# Supplementary material for: Protective Effect of Psoralea corylifolia L. Seed Extract against Palmitate-Induced Neuronal Apoptosis in PC12 Cells
Source: Evid Based Complement Alternat Med. 2016 Oct 23;2016:5410419. doi: 10.1155/2016/5410419 (PMC5097809; doi:10.1155/2016/5410419)
Supplement: Supplementary file 1 — Supplementary Figure 1 showed a correlation between reactive oxygen species production and autophagic activity in PA-treated PC12 cells. Supplementary Figure 2 showed the effects of PCE single compounds on PA-induced toxicity of PC12 cells. [file 5410419.f1.docx]

**Supplementary Materials**

**
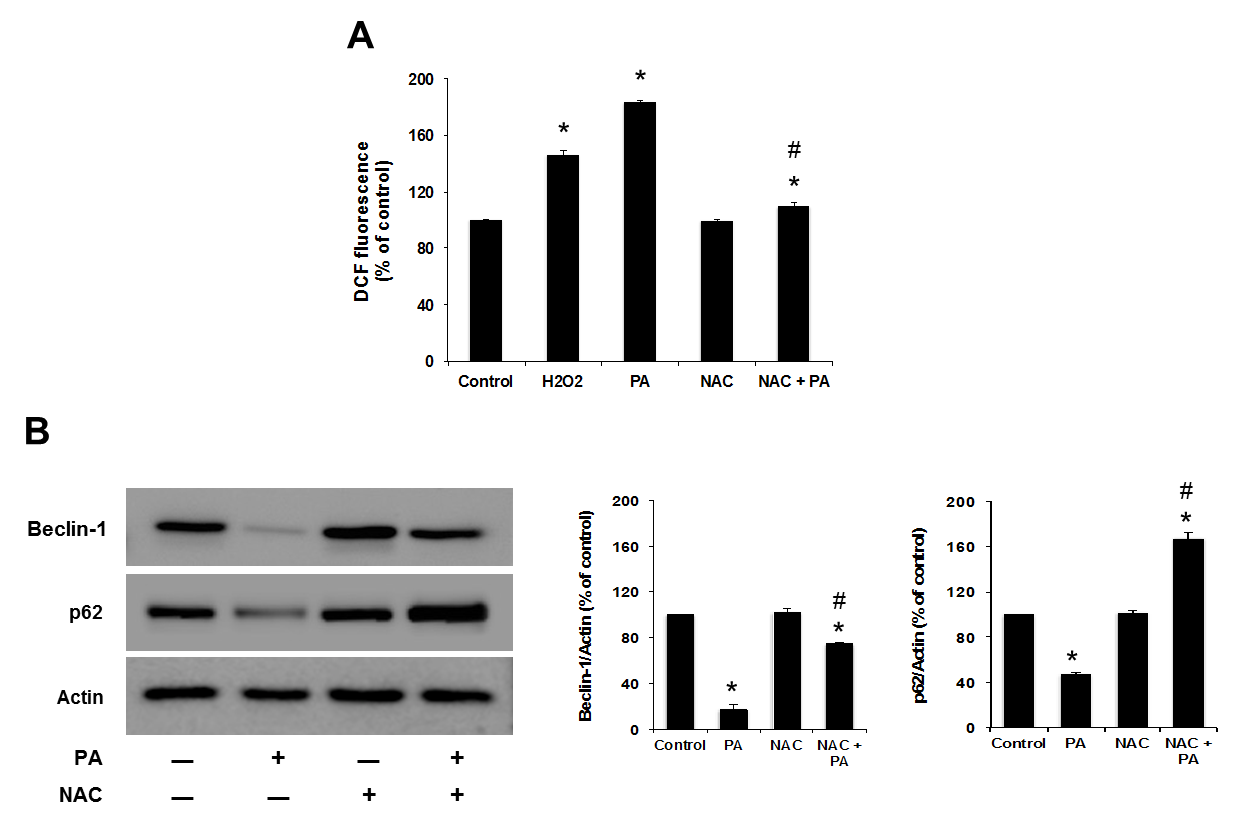
**

**Figure S1. Correlation on reactive oxygen species production and autophagic activity in PA-treated PC12 cells.**

(A) PC12 cells were pre-treated with 0.5 mM NAC for 30 min, followed by exposure to 0.4 mM PA for 3 h. The cells were stained with 10 μM H_2_-DCFDA, and intracellular reactive oxygen species generation was determined by DCF. (B) The cells were pre-treated with 0.5 mM NAC for 2 h, followed by exposure to 0.4 mM PA for 24 h. Expression levels of beclin-1 and p62 were analyzed by western blot analyses. Actin was used as the internal control. The bands were quantified by Image J software. The results represent the mean ± SEM from triplicate experiments. ^*^*P*<0.001 as compared with control. ^#^*P*<0.001 as compared with PA.


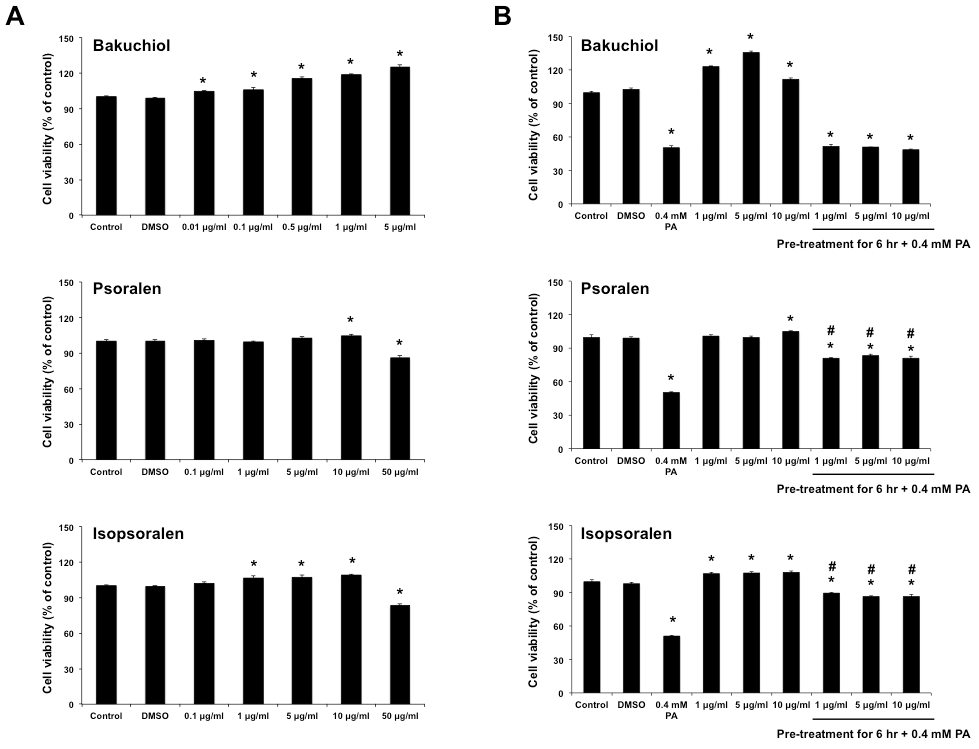


**Figure S2. Effects of PCE single compounds on PA-induced toxicity of PC12 cells.**

(A) PC12 cells were incubated in media containing bakuchiol (0.01-5 μg/ml), psoralen (0.1-50 μg/ml), or isopsoralen (0.1-50 μg/ml) for 24 h. (B) PC12 cells were pre-treated with the indicated concentrations of bakuchiol, psoralen or isopsoralen for 6 h, followed by exposure to 0.4 mM PA for 24 h. Cell viability was measured by MTT assay. The results shown represent the mean ± SEM from triplicate experiments. ^*^*P*<0.05 as compared with control. ^#^*P*<0.001 as compared with PA.
